# Low genetic diversity among *Francisella*-like endosymbionts within different genotypes of *Hyalomma dromedarii* ticks infesting camels in Saudi Arabia

**DOI:** 10.14202/vetworld.2020.1462-1472

**Published:** 2020-07-28

**Authors:** Haitham Elbir, Faisal Almathen, Ayman Elnahas

**Affiliations:** 1 Camel Research Center, King Faisal University, 400 Al-Hasa, 31982, Saudi Arabia; 2 Department of Veterinary Public Health and Animal Husbandry, College of Veterinary Medicine, King Faisal University, 400 Al-Hasa, 31982, Saudi Arabia; 3 Department of Clinical Science, College of Veterinary Medicine, King Faisal University, 400 Al-Hasa, 31982, Saudi Arabia

**Keywords:** camel, endosymbionts *Francisella* typing, *Hyalomma dromedarii*

## Abstract

**Background and Aim:**

*Hyalomma dromedarii* ticks are vectors of disease agents and hosts of *Francisella*-like endosymbionts (FLEs). Knowledge about intraspecific genetic variation among *H. dromedarii* and its *Francisella* species is limited. The aims of this study were to investigate whether certain *H. dromedarii* genotypes are specialized in carrying specific *Francisella* species genotypes and scrutinize the population structure of *H. dromedarii* ticks in Saudi Arabia.

**Materials and Methods:**

We collected 151 *H. dromedarii* ticks from 33 camels from 13 locations in Saudi Arabia. The second internal transcribed spacer (ITS2), cytochrome c oxidase subunit-1(COI), and 16S rRNA genes were used for single- and multi-locus sequence typing and phylogenetic analyses. *H. dromedarii*-borne *Francisella* was screened using the *tul4* gene and 16S rRNA *Francisella*-specific primers followed by amplicon Sanger sequencing.

**Results:**

Single-locus typing of ticks using ITS2, 16S rRNA, and COI genes yielded 1, 10, and 31 sequence types (ST), respectively, with pairwise sequence similarity of 100% for ITS2, 99.18-99.86% for COI, and 99.50-99.75% for 16S rRNA. COI sequence analysis indicated a lack of strict geographical structuration, as ST15 was found in both Saudi Arabia and Kenya. In contrast, multilocus sequence typing resolved 148 *H. dromedarii* ticks into 39 genotypes of ticks and three genotypes of FLEs. The ST2-FLE genotype was carried by the tick genotype ST35, while the ST1-FLE genotype and 41.89% of the ST3-FLE genotype were carried by the tick genotype ST32. Accordingly, there appeared to be no specialization of certain tick genotypes to harbor-specific FLE genotypes.

**Conclusion:**

For the 1^st^ time, we have provided an overview of the population structure of *H. dromedarii* ticks and FLE strains. We found a low level of genetic diversity among FLEs and non-specialized circulation of FLEs among *H. dromedarii* ticks.

## Introduction

*Hyalomma dromedarii* are ticks that infest camels and a wide range of other ungulate animals, including cattle and sheep [1,2]. The geographical distribution of *H. dromedarii* extends from North, Northwest, Central, and East Africa to the Middle East and Central and South Asia [3]. It is the most commonly reported tick seen attached to camels in Saudi Arabia [4]. *H. dromedarii* harbors several bacterial, viral, and parasitic pathogens that can infect humans and other animals. For example, in Saudi Arabia, *H. dromedarii* harbors Sindbis virus, Chick Ross virus, Kadam virus, and Alkhurma hemorrhagic fever virus; it is, therefore, suspected to play a role in the epidemiology of these pathogens [5]. In Iran, [6] molecular evidence suggests that Crimean-Congo hemorrhagic fever virus is present in *H. dromedarii*. Recently, in Pakistan [7], *Theileria annulata* parasites were found in *H. dromedarii* ticks. Notably, it is anticipated that new pathogens will emerge. As well as pathogenic microbes, *H. dromedarii* carries non-pathogenic microbes and probably endosymbiont organisms, such as *Francisella-*like endosymbionts (FLEs) [8,9]. A recent study of *H. aegyptium* ticks showed that *Francisella* spp. were significantly more abundant than other bacteria [10]. Furthermore, many symbiotic bacteria in ticks are known to interact with other microbes, affecting the competence of vectors and the ability of microbes to colonize and persist within ticks [11,12]. Hence, investigating the prevalence and diversity of FLEs in ticks is of great interest.

Intraspecies genetic analyses of *H. dromedarii* are important for defining its population structure. Different tick genotypes may differ in their pathogen colonization and competence. The veterinary and medical importance of this vector emphasizes the need for global phylogenetic studies to advance our knowledge of the relationship between different tick genotypes and their microbiota. To date, at the global level, few molecular studies have been performed to examine the intraspecies genetic diversity of *H. dromedarii*; those studies that have been performed were limited either by small sample sizes or the number of loci tested. One of these studies, in Kenya, revealed the presence of cryptic hybridization in *H. dromedarii* ticks [13]. In another study, *H*. *dromedarii* samples from Egypt were genotyped by sequence analysis of 18S rDNA, the cytochrome c oxidase subunit-1 (COI) gene, and 16S rDNA [14,15]. In India, *H. dromedarii* ticks were identified by the COI gene; further characterization and establishment of their phylogenetic status were achieved by sequencing the calreticulin gene and the second internal transcribed spacer (ITS2) [16]. Despite the importance of *Francisella* species and *H. dromedarii* ticks, there is limited information about intraspecies genetic diversity among *H. dromedarii* and their associated *Francisella* species globally and in Saudi Arabia specifically.

In this study, we attempted to address these issues by performing sequence analysis of the *Francisella* 16S rDNA gene, which has been shown to be informative for population analyses of FLEs [17]. For ticks, we selected the most commonly used genetic markers (the COI gene, ITS2, and 16S rDNA sequences) to facilitate global comparisons between geographically distant regions. The aims of this study were to investigate whether certain *H. dromedarii* genotypes are specialized in carrying specific *Francisella* species genotypes and scrutinize the population structure of *H. dromedarii* ticks in Saudi Arabia.

## Materials and Methods

### Ethical approval

Ethical approval is not necessary for this study.

### Tick collection and DNA preparation

**Figure-1:**
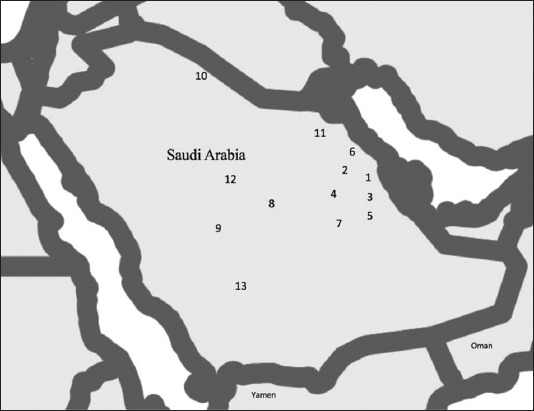
Collection sites of *Hyalomma* ticks in this study indicated by numbers. Map generated using R software version 3.6.2. 1 – Hofuf; 2 – North of Hofuf; 3 – South of Hofuf; 4 – Al Gharbia; 5 – Al Ahsa; 6 – Uqair; 7 – Khurais; 8 – Al Riyadh; 9 – Afif; 10 – Arar; 11 – Dammam; 12 – Buraidah; 13 – Asir.

One hundred and fifty-one adult ticks, obtained from 33 camels (20 males and 13 females), were collected from 13 locations in Saudi Arabia between April 2017 and January 2020. The locations are indicated on the map shown in Figure-1. It was not possible to collect the same number of samples from each location as sampling was dependent on the availability of ticks in the region. Ticks were removed from camels by hand and stored in sterile 50 ml Falcon™ conical centrifuge tubes. Ticks were placed in separate tubes according to their attachment site on a camel. The tubes were stored at −20°C until whole tick DNA was extracted using a DNeasy Blood and Tissue Kit (Qiagen, Hilden, Germany), according to the manufacturer’s instructions. Extracted DNA was stored at −20°C until being used for polymerase chain reaction (PCR) amplification and sequencing.

### Tick genotyping

PCR amplification of ITS2 of the rRNA gene and two mitochondrial genes, COI and 16S rRNA, was performed, using both specifically designed primers and previously published primers (Table-1) [18-20]. *H. dromedarii* DNA (2 μl) and 0.3 μl of each primer (10 pmol) (Macrogen, Seoul, South Korea) were added to the PCR mixture, containing one unit of Max Taq DNA Polymerase (Vivantis Technologies, Malaysia), 5 μl 10× ViBuffer (Vivantis Technologies, Malaysia), and 2 μl dNTPs (10 mM). The volume was adjusted to 25 μl by adding distilled water. Thermal cycling was performed on a TPersonal Thermocycler (BIOMETRA, Germany) with an initial 15 min cycle at 95°C, followed by 35 cycles consisting of 30 s at 94°C, 1 min at 55°C or 60°C depending on the primers, and 1 min at 72°C, followed by a final 10 min extension step at 72°C. To rule out DNA or amplicon contamination, Molecular Grade Water was used as a negative control throughout each step of the protocol. The PCR amplicons were sequenced by Macrogen Inc. (Seoul, South Korea) using BigDye (Applied Biosystems, California, USA) on an ABI3730XL DNA sequencer (Applied Biosystems, California, USA).

**Table-1:** List of Primers utilized in PCR amplification and sequencing of genes.

Primers	Sequence 5-3	References
16S-F 16S-R	CCGGTCTGAACTCAGATCAAGT GCTCAATGATTTTTTAAATTGCTGT	[18]
Cox1F Cox1R	GGAACAATATATTTAATTTTTGG ATCTATCCCTACTGTAAATATATG	[19]
ITS2-F ITS2-R	ACATTGCGGCCTTGGGTCTT TCGCCTGATCTGAGGTCGAC	[20]
ITS2-450_850 _F ITS2-450_850_R	TGT TTG CGC ATG TTG CTA TT AAT AAA TTA GCG GGG CGA CT	This study
ITS2-720F	GGC GTT CCG TCG TAG TCC	This study

PCR=Polymerase chain reaction

### *Francisella* detection and genotyping

The detection of *Francisella* DNA in individual ticks was performed through PCR of the total DNA of individual ticks, using the 16S rRNA *Francisella-* specific primers Fr153F0.1 (5-GCC CATTTGAGGGGGATACC-3) and Fr1281R0.1 (5-GGACTAAGAGTACCTTTTTGAGT-3) [21], as follows: *H. dromedarii* DNA (3 μl) and 0.3 μl of each primer (10 pmol) (Macrogen Inc., South Korea) were added to the PCR mixture, which contained one unit of Max Taq DNA Polymerase (Vivantis Technologies, Malaysia), 5 μl 10X ViBuffer (Vivantis Technologies, Malaysia), and 2 μl dNTPs (10 mM). The volume was adjusted to 25 μl by adding distilled water. Thermal cycling was performed on a TPersonal Thermocycler (BIOMETRA, Germany) with an initial 15 min cycle at 95°C, followed by 35 cycles consisting of 30 s at 94°C, 1 min at 55°C, and 1 min at 72°C, followed by a final 10 min extension step at 72°C. Molecular Grade Water was used as a negative control throughout each step of the protocol to rule out DNA or amplicon contamination. PCR product purification and sequencing were performed by Macrogen Inc. using BigDye (Applied Biosystems, California, USA) on an ABI3730XL DNA sequencer (Applied Biosystems, California, USA). The obtained sequences were blasted against the NCBI non-redundant (nr) database to identify the species they were closest to.

PCR amplification of *Francisella* 17 kDa membrane lipoprotein (*tul4*) was generated using the primers designed herein: tul4HdF (5-CTAATCCCGAAATAATATTGATAGGT-3) and tul4HdR (5-CAGTTGCCCAAGTCTTATCATTC-3). The PCR parameters used were the same as those used for the 16S rRNA *Francisella* PCR protocol used in the current study.

### Data collection and sequence analysis

Sequences of *tul4*, ITS2, COI, and 16S rRNA of *H. dromedarii* were retrieved from GenBank and compared with the sequences obtained in the present study. The nucleotide sequences were assembled and edited in CLC Main Workbench 8 software (CLC bio, Aarhus, Denmark). Sequences of the same gene were aligned using the algorithm in CLC Main Workbench 8 with default parameters. All sequences of the same gene were trimmed to the same length to enable comparisons. The ITS2, COI, and 16S rRNA nucleotide sequences analyzed herein were concatenated. Each particular sequence of COI, ITS2, and the 16S rRNA gene, in addition to the concatenated sequence, was assigned a sequence type (ST) number. The marker discrimination power of the DNA markers, COI, ITS2, and the16S rRNA gene, was calculated using the Hunter–Gaston index [22].

Phylogenetic reconstruction was performed separately for the ITS2, COI, and 16S rRNA gene sequences. In addition, relationships among *H. dromedarii* ticks sequenced herein and *H. dromedarii* ITS2, COI, and 16S rRNA sequences of *H. dromedarii* ticks obtained from GenBank were inferred using Bayesian phylogenetic analyses in MrBayes v3.2.6, as follows: Nucleotide sequences were first aligned using MUSCLE software. Bayesian phylogenetic analyses were then performed using MrBayes v3.2.6 [23] with a Jukes–Cantor model of nucleotide substitution. Sampling was performed using three independent runs (each having one cold chain and three heated chains), which were run for 1,000,000 generations.

### Nucleotide sequence accession numbers

All sequences are available through GenBank. For the COI gene sequence, the accession numbers are MH590861 to MH590886; for 16S RNA, the accession numbers are MH569476 to MH569481; and for ITS2, the accession number is MH571755.

## Results

### *Hyalomma* species

Of 151 ticks (engorged and non-engorged) collected from 33 camels (20 males and 13 females), 148 (98.01%) were *H. dromedarii*, 2 (1.32%) were *H. impeltatum*, and 1 (0.66%) was *Hyalomma* spp. Tick occurrences per camel were rare due to the use of acaricides. The ticks were mainly found attached to the brisket, axilla, lower eyelid, jaw, base of tail, and perineum region. The two *H. impeltatum* ticks were removed from the base of the tail of a female camel in Afif (Figure-1), while the single *Hyalomma* spp. was removed from the base of the tail of a female camel in Riyadh (Figure-1). *H. dromedarii* ticks were recovered from all attachment sites mentioned above.

### Genotyping of ticks

For all PCR experiments, the 148 DNA samples were efficiently amplified by PCR using the three genetic markers, and the negative controls remained negative. The lengths of the assembled sequences after trimming low-quality sequences were 722 bp for CO1 and 401 bp for 16Sr RNA, making it easy to obtain accurate sequences using the primer pairs COX-F/COX-R and 16SrRNA-F/16SrRNA, respectively. However, for ITS2, we failed to assemble high-quality sequences using ITS2-F/ITS-2R primers only. Specific internal primers (480-F, 480-R, and 720-F) were, therefore, designed, enabling assembly of a 1340 bp sequence. The genotyping of 148 *H. dromedarii* tick isolates based on concatenated sequences of COI and 16S rRNA yielded 39 STs, named ST1–ST39. The genotype ST32 represented 42.57% (63 of 148) of samples (Table-2). Individual DNA sequence analysis of ITS2, 16S rRNA, and the COI gene yielded 1, 10, and 31 genotypes, respectively (Table-3). While the concatenation of 16S rRNA and COI markers yielded a discrimination index of 0.825, this index was 0.6682 for COI, 0.3252 for 16S rRNA, and 0.0 for ITS2. Pairwise sequence comparison of the 148 *H. dromedarii* tick isolates yielded similarities of 99.18-99.86% and 99.50-99.75 for COI and 16S rRNA sequences, respectively. Alignment of COI sequences revealed a total of 33 single-nucleotide polymorphisms (SNPs); of these 33 SNPs, 27 resulted in amino acid residue changes, without any deletions or insertions. For the 16S rRNA sequences, nine SNPs were found, with no deletions or insertions. Pairwise sequence comparison of ITS2 sequences for *H. dromedarii* revealed identical sequences.

**Table-2:** Prevalence of tick genotypes based on concatenation of COI, 16S RNA gene sequence. Yellow colour indicates that the tick genotype was found in this region. White colour indicates absence of tick genotype in this region.

Genotype	Prevalence	Site 1-2-3	Site 4	Site 5	Site 6	Site 7	Site 8	Site 9	Site 10	Site 11	Site 12	Site 13
ST32	42.57											
ST10	11.49											
ST29	8.11											
ST8	3.38											
ST21	3.38											
ST33	3.38										
ST13	2.03											
ST1	1.35											
ST14	1.35											
ST15	1.35											
ST16	1.35											
ST36	1.35											
ST39	1.35											
ST2	0.68											
ST3	0.68											
ST4	0.68											
ST5	0.68											
ST6	0.68											
ST7	0.68											
ST9	0.68											
ST11	0.68											
ST12	0.68											
ST17	0.68											
ST18	0.68											
ST19	0.68											
ST20	0.68											
ST22	0.68											
ST23	0.68											
ST24	0.68											
ST25	0.68											
ST26	0.68											
ST27	0.68											
ST28	0.68											
ST30	0.68											
ST31	0.68												
ST34	0.68											
ST35	0.68											
ST37	0.68											
ST38	0.68											

**Table-3:** Performance of COI, 16S RNA, and ITS2 DNA marker used for genotyping of 62 *H. dromedarii* ticks.

DNA marker	Genotype	Prevalence of genotype %	Genotype	Prevalence of genotype %	Genotype	Prevalence of genotype %	Genotype	Prevalence of genotype %	Genotype	Prevalence of genotype %
				
	Tick COI		Tick 16S rRNA		Tick ITS2		*Francisella tul4*		*Francisella* 16S rRNA	
	ST3	0.67	ST2	0.68	ST1	100	ST1	0.67	ST1	100
	ST4	0.67	ST3	0.68			ST2	0.67		
	ST5	0.67	ST5	0.68			ST3	98.66		
	ST7	0.67	ST8	0.68						
	ST9	0.67	ST9	0.68						
	ST10	0.67	ST4	1.35						
	ST14	0.67	St10	2.03						
	ST15	0.67	ST7	3.38						
	ST16	0.67	ST1	8.11						
	ST17	0.67	ST6	81.76						
	ST19	0.67						
	ST20	0.67						
	ST21	0.67						
	ST22	0.67						
	ST23	0.67						
	ST24	0.67						
	ST25	0.67						
	ST28	0.67						
	ST29	0.67						
	ST31	0.67						
	ST1	1.34						
	ST12	1.34						
	ST27	1.34						
	ST30	1.34						
	ST2	2.01						
	ST11	2.01						
	ST13	2.69						
	ST6	3.36						
	ST18	3.36						
	ST8	11.41						
	ST26	56.38						

### *Francisella* 16S rRNA gene sequence analysis

In all PCR experiments, the 148 DNA samples efficiently PCR amplified using *Francisella*-specific primers and negative controls remained negative. An approximately 1000 bp fragment of *Francisella* 16S rRNA gene was obtained after trimming low-quality sequences, whereas all 148 16S rRNA gene sequences were identical. A comparison of *Francisella* 16S rRNA gene sequences with data available in GenBank showed 100% similarity to FLE of *H. dromedarii* (KY469285), 99.75% similarity to FLE of *H*. *dromedarii* (KY274572.1), 98.77% similarity to FLE of *Dendrobates auratus* (JQ764629.1), and 98.68%, 98.59%, and 98.30% similarity to FLE of *Ornithodoros moubata* (AB001522.1), FLE of *Hyalomma asiaticum* (KX852466.1), and *Francisella hispaniensis* (CP018093.1), respectively. Notably, similarity to *Francisella tularensis* strains varied from 98.11% to 97.9%. Accordingly, *Francisella* spp. detected herein was classified as an FLE of *H. dromedarii*. As for *Francisella* genotyping, the analysis of the 16S rRNA gene sequences derived from the 148 *Francisella* DNA samples from ticks yielded one FLE ST, which we named FLE-ST1. Similarly, the FLE of *H. dromedarii* (KY469285) usually found in Egypt had 16S rRNA gene sequences different from that of FLE-ST1 and was herein named as FLE-ST2.

### *Francisella tul4* gene sequence analysis

A comparison of *Francisella* *tul4* gene sequences with data available in GenBank showed 97.93% similarity to FLE of *Ornitnodoros porcinus* (AY375423.1), 97.58% similarity to FLE of *Dermacentor albipictus* (GU968877.1), 96.89% similarity to FLE of *Dermacentor andersoni* (AY375412.1), and 95.35% and 87.27% similarity to FLEs of *Amblyomma dubitatum* (EU441945.1) and *F. tularensis* subsp. *tularensis* (CP003049.2), respectively.

Alignment of the *tul4* gene sequence showed two SNPs. The genotyping of 148 *H. dromedarii* tick isolates based on the *tul4* gene yielded three STs, named FLE-*H. dromedarii* ST1-ST3 (Table-4). The genotype FLE-*H. dromedarii* ST-3 represented 98.6% (146 out of 148 samples), followed by genotype FLE-*H. dromedarii* ST1 (0.7%), which was found in the Buraidah region, and FLE-*H. dromedarii* ST2 (0.7%), which was found in the Asir region (Figure-1).

**Table-4:** Genotypes of ticks and their associated genotypes of FLEs.

Tick sample	Tick genotype	FLEs genotype
2	ST18	ST3-FLE
3	ST32	ST3-FLE
4	ST21	ST3-FLE
5	ST32	ST3-FLE
6	ST5	ST3-FLE
7	ST13	ST3-FLE
8	ST26	ST3-FLE
9	ST32	ST3-FLE
10	ST14	ST3-FLE
11	ST13	ST3-FLE
13	ST32	ST3-FLE
14	ST15	ST3-FLE
15	ST32	ST3-FLE
16	ST10	ST3-FLE
17	ST32	ST3-FLE
20	ST17	ST3-FLE
23	ST32	ST3-FLE
24	ST1	ST3-FLE
25	ST10	ST3-FLE
26	ST15	ST3-FLE
27	ST32	ST3-FLE
28	ST14	ST3-FLE
29	ST6	ST3-FLE
30	ST1	ST3-FLE
31	ST32	ST3-FLE
32	ST32	ST3-FLE
33	ST32	ST3-FLE
34	ST11	ST3-FLE
35	ST31	ST3-FLE
36	ST28	ST3-FLE
37	ST8	ST3-FLE
38	ST24	ST3-FLE
39	ST32	ST3-FLE
40	ST19	ST3-FLE
41	ST10	ST3-FLE
43	ST32	ST3-FLE
44	ST30	ST3-FLE
45	ST32	ST3-FLE
46	ST29	ST3-FLE
47	ST12	ST3-FLE
48	ST29	ST3-FLE
49	ST32	ST3-FLE
50	ST2	ST3-FLE
51	ST21	ST3-FLE
52	ST32	ST3-FLE
53	ST13	ST3-FLE
54	ST32	ST3-FLE
55	ST32	ST3-FLE
56	ST7	ST3-FLE
57	ST32	ST3-FLE
59	ST22	ST3-FLE
60	ST9	ST3-FLE
62	ST32	ST3-FLE
63	ST32	ST3-FLE
65	ST27	ST3-FLE
66	ST32	ST3-FLE
67	ST32	ST3-FLE
68	ST23	ST3-FLE
70	ST32	ST3-FLE
71	ST32	ST3-FLE
72	ST3	ST3-FLE
73	ST32	ST3-FLE
100	ST32	ST3-FLE
101	ST29	ST3-FLE
103	ST32	ST3-FLE
104	ST32	ST3-FLE
105	ST10	ST3-FLE
106	ST29	ST3-FLE
109	ST32	ST3-FLE
110	ST32	ST3-FLE
111	ST29	ST3-FLE
112	ST36	ST3-FLE
113	ST36	ST3-FLE
114	ST29	ST3-FLE
115	ST29	ST3-FLE
116	ST29	ST3-FLE
117	ST10	ST3-FLE
118	ST32	ST3-FLE
119	ST33	ST3-FLE
120	ST32	ST3-FLE
121	ST32	ST3-FLE
122	ST32	ST3-FLE
123	ST10	ST3-FLE
124	ST10	ST3-FLE
125	ST33	ST3-FLE
126	ST32	ST3-FLE
127	ST32	ST3-FLE
128	ST32	ST3-FLE
129	ST33	ST3-FLE
130	ST29	ST3-FLE
131	ST32	ST3-FLE
132	ST10	ST3-FLE
133	ST10	ST3-FLE
134	ST10	ST3-FLE
135	ST10	ST3-FLE
136	ST32	ST3-FLE
137	ST10	ST3-FLE
139	ST21	ST3-FLE
140	ST33	ST3-FLE
141	ST21	ST3-FLE
142	ST29	ST3-FLE
143	ST10	ST3-FLE
144	ST10	ST3-FLE
145	ST21	ST3-FLE
146	ST32	ST3-FLE
147	ST33	ST3-FLE
148	ST32	ST3-FLE
149	ST32	ST3-FLE
150	ST32	ST3-FLE
151	ST32	ST3-FLE
152	ST29	ST3-FLE
154	ST37	ST3-FLE
155	ST29	ST3-FLE
156	ST32	ST3-FLE
157	ST10	ST3-FLE
158	ST32	ST3-FLE
159	ST32	ST3-FLE
160	ST4	ST3-FLE
164	ST32	ST3-FLE
165	ST32	ST3-FLE
167	ST20	ST3-FLE
168	ST25	ST3-FLE
170	ST32	ST3-FLE
171	ST32	ST3-FLE
172	ST32	ST3-FLE
173	ST34	ST3-FLE
174	ST16	ST3-FLE
175	ST32	ST3-FLE
176	ST32	ST3-FLE
177	ST32	ST3-FLE
178	ST8	ST3-FLE
179	ST32	ST3-FLE
180	ST8	ST3-FLE
181	ST8	ST3-FLE
182	ST32	ST3-FLE
183	ST8	ST3-FLE
184	ST10	ST3-FLE
185	ST32	ST1-FLE
186	ST10	ST3-FLE
187	ST16	ST3-FLE
188	ST35	ST2-FLE
189	ST38	ST3-FLE
190	ST39	ST3-FLE
191	ST32	ST3-FLE
192	ST39	ST3-FLE
193	ST32	ST3-FLE
194	ST32	ST3-FLE
196	ST32	ST3-FLE

FLE=*Francisella*-like endosymbionts

### Phylogenetic analysis

**Figure-2:**
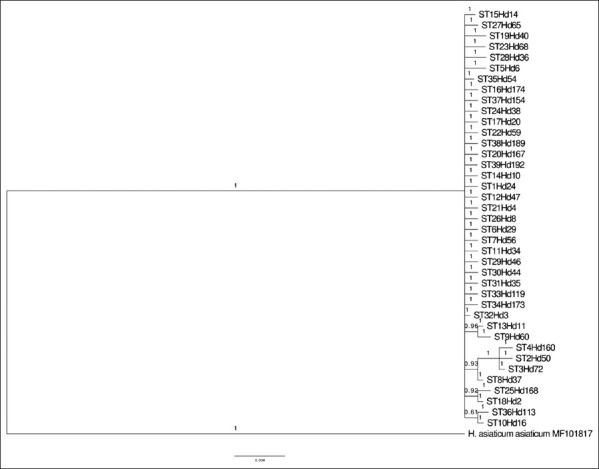
Bayesian Markov chain Monte Carlo analysis based on concatenation of 16S rDNA and cytochrome c oxidase subunit-1 sequences for 148 *Hyalomma dromedarii*. Numbers represent posterior probabilities.

Consultation of the GenBank database revealed that *H. dromedarii* sequences comprised 14 16S rRNA nucleotide sequences, from Senegal, Pakistan, Egypt, Saudi Arabia, and Iraq. There were 22 COI nucleotide sequences, from Kenya, India, United Arab Emirates, Ethiopia, Senegal, Pakistan, Egypt, Saudi Arabia, and Iraq. ITS2 comprised 15 nucleotide sequences, from India, Egypt, Pakistan, Iraq, Saudi Arabia, Iran, and Iraq. Not all sequences retrieved from GenBank were included in this analysis; short sequences were excluded. The concatenation of the 16S rDNA and COI sequences of *H. dromedarii* separated the strains into different clades, as shown in Figure-2.

**Figure-3:**
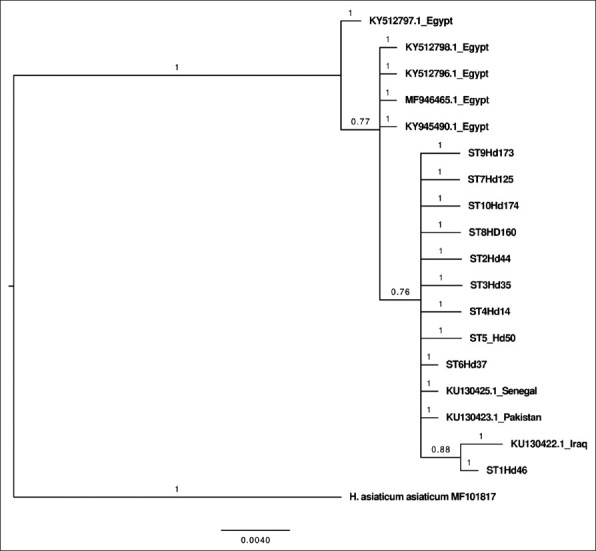
Bayesian Markov chain Monte Carlo analysis based on combining sequence types of 16S rRNA sequences in this study, with GenBank 16S rRNA sequence. Numbers represent posterior probabilities.

**Figure-4:**
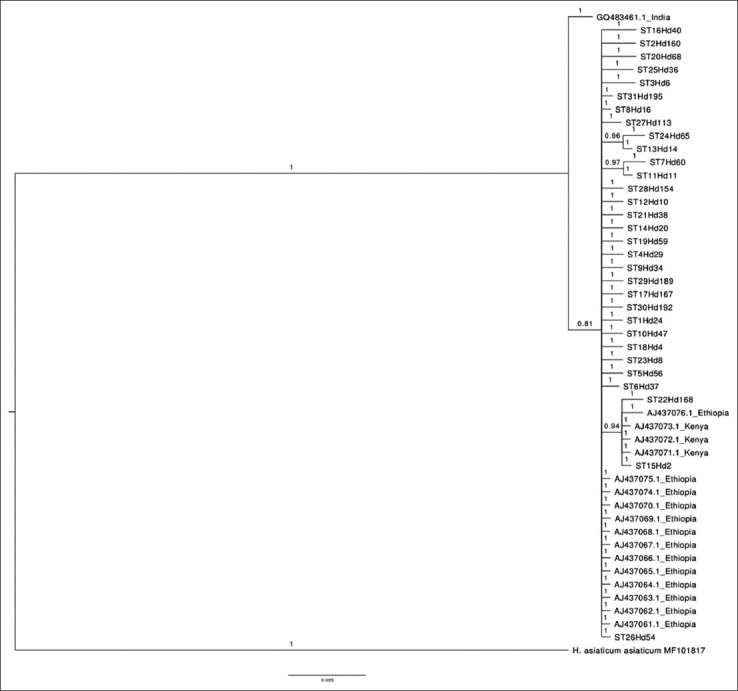
Bayesian Markov chain Monte Carlo analysis based on combining sequence types of cytochrome c oxidase subunit-1 (COI) sequences found here in ticks, with GenBank COI sequence. Numbers represent posterior probabilities.

A phylogenetic tree combining the STs of 16S rRNA sequences obtained in this study with GenBank 16S rRNA sequences yielded three clades: Clades A and B, consisting of isolates from Egypt and clade C, consisting of Saudi Arabia *H. dromedarii* isolates clustered with ticks from Senegal, Iraq, and Pakistan (Figure-3). The phylogenetic analysis combining STs of COI sequences found in ticks in our study with GenBank COI sequences revealed an absence of strict geographical structuration since genotype ST15 was found in both Saudi Arabia and Kenya (Figure-4).

**Figure-5:**
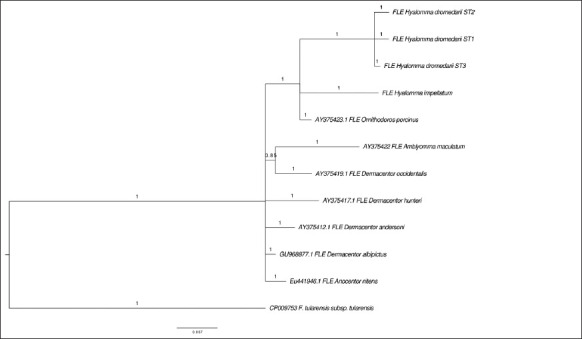
Bayesian Markov chain Monte Carlo analysis based on combining sequence types of tul4 sequences found here in ticks, with GenBank tul4 sequence. Numbers represent posterior probabilities.

For FLEs of *H. dromedarii*, the phylogenetic tree inferred using the *tul4* sequences clearly separated FLEs of *Hyalomma* strains from the FLEs of other ticks (Figure-5).

## Discussion

The multityping approach used in this study revealed a high degree of genetic diversity among *H. dromedarii* collected from camels in Saudi Arabia. Even though we examined a relatively small set of *H. dromedarii*, we observed a high diversity index among these ticks, since 148 *H. dromedarii* ticks yielded 39 genotypes, as determined by the concatenation of COI and 16S rRNA gene sequences. The use of several genetic markers offers better resolution over the use of a single genetic marker, as has been used previously [14,15]. Our results revealed that COI sequences were more variable, in contrast with the low nucleotide variation seen in 16S rRNA and the highly conserved ITS2 sequence. These findings were consistent with those of previous tick studies [24-26]. Consequently, we concluded that COI is a more informative marker than the 16S rRNA gene for studying intraspecific diversity, whereas the ITS2 sequence is not informative but is suitable for *H. dromedarii* identification.

The tick genotype ST32 represented the dominant genotype prevalent in 11 out of 13 locations, which suggests the circulation of this genotype throughout Saudi Arabia. Plausible explanations for this could be that the ST32 genotype is well adapted to persist in its camel host or the frequent movement of camels between different locations as they seek pasture. Furthermore, ST32 showed no preference for a specific attachment site and was found widely distributed around a camel’s body.

A 16S rRNA phylogenetic tree showed that *H. dromedarii* ticks of Saudi Arabia were clustered with ticks from Senegal, Iraq, and Pakistan. Moreover, COI phylogenetic analysis revealed an absence of strict geographical structuration, as genotype ST15 was found in both Saudi Arabia and Kenya. A reasonable explanation for this could be the longstanding trade in camels between these countries, which might play a significant role in spreading ticks. More *H. dromedarii* samples are, therefore, needed to shed light on the global diversity of this species.

In this study, a total of 148 ticks were screened for *Francisella* species. *F. tularensis* was not detected in any samples. In contrast, FLEs were highly prevalent (100%) in *H. dromedarii*. These results indicate some sort of beneficial relationship between FLEs and *H. dromedarii*. The previous studies into *H. dromedarii* showed differences in the prevalence rates of FLEs, which varied from 6% to 89.8% [8,9]. A possible explanation for this discrepancy in results could be explained either by FLEs being present but undetectable due to low concentrations of DNA or that certain genotypes of *H. dromedarii* ticks have no capacity to harbor FLEs.

The presence of different STs of FLEs of *D. andersoni* and *D. variabilis* has been reported in Canada following the sequencing of 16S rRNA [17]. In addition, the FLE-ST2 genotype has been reported in Egypt. Here, we observed no intraspecies discrimination among Saudi Arabian FLE strains using 16S rRNA genes, in contrast with the *tul4* gene, which grouped the 148 FLEs of *H. dromedarii* into three genotypes. These findings indicate a low level of genetic diversity among the FLE population of *H. dromedarii*. Indeed, the diversity of FLEs in *H. dromedarii* did not overlap with that of *H. dromedarii*. The ST2-FLE was carried by the tick genotype ST35, while the ST1-FLE genotype and 41.89% of the ST3-FLE genotype were carried by the tick genotype ST32. Accordingly, these results imply that, within *H. dromedarii*, there is no specialization of a certain tick genotype for their capacity to harbor-specific genotypes of *H. dromedarii* FLEs.

## Conclusion

The tick *H. dromedarii* is the dominant tick species infesting camels in Saudi Arabia. The high prevalence of FLEs in *H. dromedarii* suggests that *Hyalomma* and FLEs have some sort of symbiotic relationship. Although FLEs are spread throughout all *H. dromedarii*, the low diversity of FLEs did not overlap with the high diversity of *H. dromedarii*. The ST2-FLE was carried by the tick genotype ST35, while the ST1-FLE genotype and 41.89% of the ST3-FLE genotype were carried by the tick genotype ST32. Accordingly, these results imply that, within *H. dromedarii*, there is no specialization of certain tick genotypes for their capacity to harbor-specific genotypes of *H. dromedarii* FLEs. Furthermore, we have increased the number of *Hyalomma* species reported to carry FLEs from five to six species, by detecting FLEs in *H. impeltatum* for the 1^st^ time.

## Authors’ Contributions

HE conducted the experiment, interpreted the results, and drafted the manuscript. FA designed the experiment and data analysis and revised the manuscript. AE collected the samples and revised the manuscript. All authors have read and approved the final manuscript.
